# Evaluating the psychometric properties of the German adaptation of the client attachment to therapist scale

**DOI:** 10.1186/s12874-022-01548-2

**Published:** 2022-04-05

**Authors:** Katja Petrowski, Bjarne Schmalbach, Joram Ronel, Ileana Schmalbach, Elisabeth Olliges

**Affiliations:** 1 Universitätsklinikum Carl Gustav Carus an der Technischen Universität Dresden, Abteilung für Innere Medizin III, Dresden, Germany; 2 Department of Medical Psychology and Medical Sociology, University Medical Center of the Johannes Gutenberg University Mainz, Mainz, Germany; 3 Department of Psychosomatic Medicine and Psychotherapy, Klinik Barmelweid AG, Barmelweid, Switzerland; 4 Department of Psychosomatic Medicine and Psychotherapy Klinikum rechts der Isar, Technische Universität München, Munich, Germany

**Keywords:** Attachment representation, Therapeutic relationship, Psychotherapy, Psychometric evaluation

## Abstract

**Background:**

The present study examines the psychometric properties of the German adaptation of the Client Attachment to Therapist Scale (CATS). The validity of the scale as originally proposed has recently been brought into question, as patients were identified as “pseudosecure”.

**Methods:**

We examined the measure’s factorial structure, as well as reliability and validity towards related measures using a clinical sample of *N* = 354 participants.

**Results:**

We found the original model, consisting of 36 items to be lacking in terms of model fit and construct validity. A shortened 12-item version exhibited markedly improved model fit and reliability. Correlations to related constructs demonstrated that none of the scale’s validity was lost by shortening it. Furthermore, we showed scalar invariance across groups of age and sex.

**Conclusions:**

The shortened CATS-S can be recommended for future use in clinical research in German-speaking populations as a valid, reliable, and economical alternative to the longer version.

## Background

Bowlby [1], postulated that the psychotherapeutic relationship resembles a behavioral repertoire comparable to attachment, providing a possible explanation of the expectations and behaviors displayed by clients in the therapeutic setting. As in a parental or primary caregiver relationship, the psychotherapist provides emotional support and regulation, accessibility, comfort, sympathy, and a “secure base” promotion explorative behaviors [2]. Therefore, nourishing a therapeutic relationship can be understood as certain form of adult attachment, which is mostly coined by past relationships experiences in the client’s childhood [1]. Hence, attachment theory models foster understanding in therapeutic environments.

Although a large body of literature has used concepts of attachment theory in psychotherapy research [3–6], the therapeutic relationship was rarely conceptualized from an attachment perspective. Subscales of existing measures—for example, the “bond” subscale of the Working Alliance Inventory (WAI) [7] — capture only one aspect of this attachment relationship. Therefore, important components of the attachment theory are missing, such as clients’ feelings and attitudes toward the counselor from an attachment perspective. Furthermore, the clients’ capacity to form positive, secure attachments towards their therapists [8], the quality of the working alliance [9], the level of basic social competencies [5, 10], and clients’ secure and insecure attachment representation [1] should be assessed.

Therefore, the Client Attachment to Therapist Scale (CATS) was developed. To this end, nine experienced therapists generated items based on the description of the behavior displayed by infants of secure, ambivalent, and avoidant attachment described by Ainsworth and her colleagues [11]. The panel generated a total of 272 items. After removing redundant items and changing the wording to minimize response set bias, the initial version of the CATS contained 100 items with a 6-point response scale. After pretesting, a factor analysis based on *n* = 138 patients revealed one large factor, which captured client perceptions of secure attachment and two smaller factors, which captured more troubled attachments to the therapist. The subscales (*Secure*, *Avoidant-Fearful* and *Preoccupied-Merger*) demonstrated acceptable internal and retest reliability (α > .72; *r > .*63).

Clients with high scores in the CATS *Secure* subscale viewed their therapists as emotionally available, supportive, and encouraging exploration of an uncomfortable emotional experiences [1]. These types of clients are prone to record a positive working alliance, good object-relations capacity, and a relatively solid perception of self-efficacy. Clients with elevated scores on the CATS *Preoccupied-Merger* subscale, long for boundaryless affiliation to the therapist and wish a deeper and intense relationship. These clients are also characterized by a constant rumination on their therapist and are highly compliant in terms of depending on others. The relationship of the CATS *Preoccupied-Merger* subscale with the working alliance implies attachment to the therapists, even without a dialectical goal settlement between client and therapist. Clients who scored high on the CATS *Avoidant-Fearful* subscale tend to mistrust their therapists, avoided rejection, and were not compliant in open up to their therapists. Clients who scored high on the CATS *Avoidant-Fearful* subscale exhibited the weakest working alliances and object-relations shortcomings.

For differential validity, the CATS was applied together with social competencies, social support, and personality questionnaires [12, 13]. Furthermore, in various psychotherapy studies the CATS was used to specify the effect of the client attachment to therapist onto transference [14], depth in session [15], interpersonal process [13], WAI, and outcome [16–18]. In addition, the premature termination [19], session impact [4], emotion, and mood awareness [20] could be predicted by the client attachment to therapist (CATS). Recent studies also showed the influence of the therapist’s attachment and the clients’ attachment to therapist onto WAI and session exploration [21–26].

Even though the CATS was psychometrically investigated, the factorial structure of the CATS has not been yet investigated by a confirmatory factor analysis. This is of special importance since, patients were recently identified as pseudosecure attached patients [27]. Patients with pseudosecure and secure attachment to the therapist present some similar features in the early sessions such as: easily bonding, readiness to self-disclose, regard their therapist in strongly positive terms, and place high value on the therapeutic relationship [27]. The crucial difference is that patients with a pseudosecurity pattern idealize their therapist and are highly dependent of him or her. This might be explicable by the high intercorrelation between the clients’ secure attachment to the therapist (CATS-*Secure*) with the clients’ avoidant attachment (CATS-*Avoidant-Fearful*; *r* = −.693, *p* < .001). Hence, one of our aims was to identify clearly separable factors and provide a clear-cut illustration of the three-factor structure of the CATS. For this aim, the German adaptation of the original scale was implemented [24]. Such was previously translated by experts from English into German according to common translation guidelines [25–28]. Nevertheless, the psychometric properties were not primary focus of the past investigation [24]. Therefore, a further aim of the present study is to evaluate the psychometric properties of the scale and if required optimize them based on a large sample. Especially in clinical research, in which patients do not feel well, long questionnaires with 36 items such as the CATS might be a tiring and a more economic version would be highly appreciated. Consequently, the need of shorter scale in this research field may be a practical contribution for patients and therapists.

## Methods

In order to ensure the quality of the analyses, two samples were collected at two different timepoints. Thus, in the first study (Study 1), the psychometric properties of the translated scale were piloted (Sample 1). With the purpose of confirmatory validation, a second study (Study 2) was conducted to evaluate the final scale (Sample 2). The results of each study are reported in the respective results section.

### Participants

The attachment representations of patients in a naturalistic inpatient were assessed. Similar to instructions given to the participants in the original CATS development study, all clients in both of our samples were reassured their therapist would never have access to ratings of their therapeutic relationship. The study was approved by the ethics committee of [masked for review] (Code of ethics: 112052007). In general, the average duration of treatment was 64.05 calendar days (*SD* = 28.54). In addition to a daily group therapy session with their primary therapist, patients also saw their primary therapist for individual focal therapy twice a week for 50 min. As a result, the contact with their primary therapists was intensified. The other members of the therapeutic team had patient contacts lasting merely 10 to 15 min. The psychotherapists who were asked to take part in our study had a professional background with a psychological or medical education with an additional license for practicing psychotherapeutic treatment.

In Study 1 (Sample 1 = *N* = 433), 79 participants were excluded due to missing data, leading to a total sample size of *N* = 354. Thirty-one participants in this sample had missing values of no more than three items in a scale. We substituted those missing values with the item mean. The imputation via mean values was carried out on a per-person-basis. Thus, the personal characteristics of each participant have been taken into account. Sample 2 (*N* = 306), no missing values were identified. The ICD-10 diagnoses confirmed by SCID [29, 30] were in Sample 1 mostly affective (F30–39; 114/32%) followed by anxiety disorders (F40-F41; 55/16%), adjustment/stress disorders (F43; 37/10%), and somatoform disorders (F45; 35/10%). In Sample 2 (*N* = 306), most of the diagnoses were mental, behavioral and neurodevelopmental (F01-F99; 98/32%) followed by factors influencing health status and contact with health services (Z00-Z99; 27/8.8%). The majority of the mental disorders were related to anxiety, dissociative, stress-related, somatoform and other nonpsychotic mental disorders (F40-F4; 15/5.0%), followed by mental and behavioral disorders due to psychoactive substance use (F10-F19; 10/3.3%) as well as behavioral syndromes associated with physiological disturbances and physical factors (F50-F59; 7/2.2%). The general symptom severity at admission was quite high (SCL-90-R-GSI T value at admission was *M* = 69.98; *SD* = 11.35) and had significantly decreased at discharge, even though the level was still high (*M* = 61.89, *SD* = 13.70) (*t* = 13.36, *df* = 335, *p* < .001; *d* = .65). Depression symptoms were also quite pronounced (BDI-score at admission was *M* = 26.61; *SD* = 12.36) and significantly decreased at the end of therapy (*M* = 16.60, *SD* = 12.72; *t* = 15.95, *df* = 285, *p* < .001; *d* = .79). The sociodemographic data of the participants is reported in Table 1.

**Table 1:** Sociodemographic characteristics of the study samples

	Sample 1 (*n* = 354)		Sample 2 (*n* = 306)	
	*n*	%	*n*	%
Gender				
Female	263	74.3	188	61.4
Male	88	24.9	118	38.6
Missing	3	.8		
Age, years	*M* = 37.24, *SD* = 13.12		*M* = 46.90, *SD* = 16.87	
Missing	7	2.0	0	0
Family status				
Single	108	3.5		
Committed Relationship	88	24.9		
Married	107	3.2		
Separated	9	2.5		
Divorced	26	7.3		
Widowed	2	.6		
Missing	19	5.4		
Education				
8–10 years	212	51.4		
≤ 12 years	111	31.4		
School for handicapped children	10	2.8		
No schooling completed	2	.6		
Student	4	1.1		
Missing	15	4.2		
Employment				
Blue-collar worker	110	31.1		
White-collar worker	131	37.0		
Self-employed	23	6.5		
Un-employed	42	11.9		
Missing	48	13.6		

#### Measures

For the study at hand (Study 1,2) we implemented the German-versions of each of the following measures.

A German adaptation of the *Client Attachment to Therapist Scale* (CATS) [5], was applied to analyze the patients’ feelings and expectations toward their therapist from an attachment point of view. The CATS encompass 36 items arranged in three sub-scales: the *Secure* sub-scale (14 items: e.g., ‘my counselor is dependable’), the *Avoidant-Fearful* sub-scale (12 items: ‘I don’t like to share my feelings with my counselor’), and the *Preoccupied-Merger* sub-scale (10 items: ‘I think I am my counselor’s favorite client’). High avoidance mirrors the patients’ distrust towards the therapist and disagreement whereby patients are not compliant in opening up and feel uncomfortable and disgusted when speaking in therapy. High preoccupation reflects the patient’s thoughts about the therapist and the desire for a closer the therapeutic relationship. Patients answer on a six-point-scale from strongly agree (1) to strongly disagree (6). The evidence of validity was evinced by significant correlations of the CATS sub-scale scores with scales of adult attachment, working alliance, and object relations. The internal reliabilities (Cronbach’s Alpha) for the *Secure*, *Avoidant–Fearful* and *Preoccupied-Merger* sub-scales were .78, .83, and .82, respectively [5]. In the current sample, the internal reliabilities (Cronbach’s Alpha) for the sub-scales ranged from .73 to .81. The characteristics of the CATS items and scales are displayed in Table 2.

**Table 2:** Characteristics of the CATS* items and scales (Sample 1)

	*M* (*SD*)	γ_1_	γ_2_	*r*_*it*_	*F1*	*F2*	*F3*
CATS 1	4.60 (1.47)	−.80	−.54	.66	.609	−.101	−.127
CATS 2 ^S^	4.60 (1.30)	−1.07	.45	.67	.579	.110	−.215
CATS 3 ^S^	5.12 (1.05)	−1.73	3.72	.66	.631	−.060	−.118
CATS 4	4.07 (1.40)	−.60	−.40	.65	.587	.105	−.207
CATS 5	4.74 (1.47)	−.99	−.17	.58	.566	−.179	−.054
CATS 6 ^S^	4.67 (1.20)	−1.20	1.40	.67	.610	.124	−.176
CATS 7	3.32 (1.72)	.20	−1.28	.22	.055	−.003	−.278
CATS 8	4.24 (1.38)	−.95	.33	.70	.559	.089	−.295
CATS 9	4.42 (1.59)	−.54	−1.00	.63	.608	−.220	−.076
CATS 10	4.25 (1.56)	−.93	−.27	.52	.565	−.097	.016
CATS 11 ^S^	3.74 (1.57)	−.51	−.93	.55	.609	.131	−.031
CATS 12	4.61 (1.20)	−1.22	1.27	.76	.637	.028	−.283
CATS 13	4.55 (1.26)	−1.11	.82	.68	.721	−.039	−.05
CATS 14	4.87 (1.43)	−1.53	1.53	.36	.417	.036	.046
CATS 15 ^S^	1.88 (1.24)	1.47	1.32	.66	−.135	.169	.610
CATS 16	2.71 (1.59)	.27	−1.36	.51	.197	.059	.727
CATS 17	1.95 (1.17)	1.71	2.92	.50	−.365	−.008	.301
CATS 18 ^S^	2.81 (1.55)	.32	−1.27	.57	.033	−.110	.691
CATS 19 ^S^	1.62 (1.10)	1.89	2.62	.62	−.093	.059	.602
CATS 20 ^S^	1.90 (1.33)	1.47	1.12	.67	−.023	.111	.683
CATS 21	1.53 (.96)	2.37	5.96	.55	−.262	.078	.397
CATS 22	2.39 (1.57)	.77	−.73	.35	.105	−.011	.487
CATS 23	3.16 (1.56)	.55	−.86	.35	−.577	−.225	.097
CATS 24	1.42 (.89)	2.70	7.66	.50	−.162	.233	.373
CATS 25	2.02 (1.39)	1.30	.59	.72	−.253	−.031	.623
CATS 26	2.56 (1.69)	.69	−.96	.48	−.344	.282	.251
CATS 27 ^S^	1.72 (1.26)	1.94	2.99	.66	−.002	.711	.038
CATS 28 ^S^	1.91 (1.38)	1.40	.75	.64	.049	.716	.037
CATS 29	1.89 (1.33)	1.42	.90	.62	−.166	.675	.111
CATS 30 ^S^	2.83 (1.57)	.24	−1.23	.58	.152	.581	.116
CATS 31	1.17 (.61)	4.55	23.41	.26	−.053	.320	−.106
CATS 32	1.20 (.65)	4.12	19.01	.39	−.028	.426	.047
CATS 33	2.01 (1.37)	1.06	−.26	.64	−.058	.740	−.127
CATS 34 ^S^	1.91 (1.36)	1.31	.40	.52	.213	.532	.075
CATS 35	1.45 (.93)	2.42	5.58	.53	.046	.551	−.042
CATS 36	1.70 (1.24)	1.78	2.18	.46	.084	.424	.276
Secure^L^	4.41 (.91)	−.75	.48				
Avoidant^L^	2.16 (.84)	.97	.85				
Preoccupied^L^	1.78 (.77)	1.24	1.75				
Secure ^S^	4.55 (1.00)	−1.00	1.04				
Avoidant ^S^	1.80 (1.02)	1.54	1.97				
Preoccupied ^S^	1.88 (1.11)	1.35	1.26				

*M* Mean, *SD* Standard deviation; γ_1_ = skewness; γ_2_ = kurtosis; *r*_*it*_ = corrected item-total correlation; ^L^ = subscales according to original CATS; ^S^ = items and subscales according to short model CATS-S. F = factor loadings. * The order in the present Table does not represent the order of the original scale

The original CATS was translated by experts into German according to the translation guidelines based on the WHO protocol of translation and adaption of instruments (e.g., forward and back translation, pre-testing and final version) [28] which is in line with the procedure of translation, back-translation, and verification of suggested protocols in the past [25–27].

Psychological distress was evaluated with the German version of the *Symptom Check List* (SCL-90-R) [31] at the time of admission and at termination of therapy. The global severity index (GSI) of the Symptom Check List was applied as a main outcome measure from the patients’ point of view. The GSI measures general symptom distress; its reliability and validity have been shown in numerous studies. A German validation study replicated the scale’s high internal consistency of .94 to .98 and a high retest-reliability of .79 to .90 [32].

*The Bielefeld Client Expectations Questionnaire* (BFCE) [33] evaluates the expectations of the patients towards the therapist. The scale covers the following dimensions: *Fear of Rejection, Readiness for Self-Disclosure,* and *Conscious Need for Care*, conveyed in 11 and 10 items, accordingly. A 5-point scale ranging from (0) = “does not apply at all” to (4) = “does fully apply”. The internal consistency is respectable (α = .83–.84) [33].

The German version of the *Helping Alliance Questionnaire* (HAQ) [34, 35] was implemented to assess the quality of the therapeutic relationship by 22 Items, which reflect the two factors relationship satisfaction and outcome satisfaction from the patient and the therapist point of view. Answer options range from (1) = “I strongly feel it is not true.” to (6) = “I strongly feel it is true.” Reliability coefficients are α = .89 for relationship satisfaction and α = .84 for outcome satisfaction [35]. An overview of the scales in provided in Table 3.

**Table 3:** Overview of the implemented scales in Study1,2

Scales	Operationalization	Dimensions
CATS^a^	patients’ feelings and expectations toward their therapist from an attachment point of view.	Secure, avoidant-fearful, preoccupied-Merger.
SCL-90-R	Psychological distress	Somatization, obsessive-compulsive, interpersonal sensitivity, depression, anxiety, hostility, phobic anxiety, paranoid ideation, psychoticism.
BFCE	Expectations of the patients towards the therapist	Fear of Rejection, readiness for self-Disclosure, and conscious need for care.
HAQ	Quality of the therapeutic relationship	Relationship satisfaction, outcome satisfaction.

^a^German adaptation of the *CATS* Client Attachment to Therapist Scale, *SCL-90-R* Symptom Check List-90-Revised, *BFCE* The Bielefeld Client Expectations Questionnaire, *HAQ* Helping Alliance Questionnaire

### Procedure

In both studies, the patients were instructed about the aim of the research project and about the data policies of the study (e.g., confidentiality clause). The participants were assured that neither their primary therapist nor third parties could have access to their submitted data. After submitting their informed consent for participation, the patients were included in the study. Thereafter, they filled out routine assessment questionnaires of symptoms at the beginning and the end of the treatment. To that end, participants were told to refer to their primary therapist when answering the CATS. At the end of the psychotherapeutic intervention (Sample 1,2: after approximately 63 days, 12 sessions), they filled out the German adaptation of the Client Attachment to Therapist (CATS) questionnaire.

#### Statistical analyses

We computed the statistical analysis using R and the packages *EFAutilities*, *lavaan*, *paran*, *semTools,* and *stuart* [36–40]. We employed an α level of .05 for tests of significance, unless noted otherwise. Our hypotheses and data-analytical plan were specified a priori. For the exploratory factor analysis (EFA), we first conducted parallel analysis (PA) [41] to establish the number of components in the data by comparing the empirical eigenvalues to those of randomly generated data sets with the same general properties. Subsequently, we applied different methods for item reduction and model generation. For these calculations, we conducted an EFA applying ordinary least squares extraction and oblique rotation. Thereafter, the item descriptive statistics were examined. Items with the following characteristics were discarded: loadings smaller than .500, cross-loadings higher than .250, item-total correlations smaller than .500, or absolute skewness and excessive kurtosis values larger than 2 or 4, respectively [42, 43], or multiple of the above-mentioned criteria. Next, we used *stuart* to further shorten the model and tested it in the confirmatory Sample 2. Stuart works with ant colony optimization to generate and evaluate subsets of a scale and maximize model fit. For this procedure we chose models with four items per scale. For validity purposes, we conducted Person Product Moment correlations between the CATS-S subscales and related psychological constructs (Table 8), as described in the section *measures*.

In Study 2, we computed a confirmatory factor analysis (CFA) with the robust weighted least square method (WLSMV in *lavaan*) [44]. To evaluate the model fit, we used the commonly recommended indices and cutoff values [45–48]: The χ^2^-statistic should ideally not be significant, but this is unlikely with larger samples [49]. The comparative fit index (CFI) should be higher than .95 for a model to be acceptable, while values higher than .97 indicate particularly good fit. Similar criteria are generally applied for the Tucker Lewis index (TLI), although this index is always lower than the CFI. The root mean square error of approximation (RMSEA) and its 90% confidence interval should be lower than .08, while values lower than .05 suggest good fit. The standardized root mean square residual (SRMR) is evaluated by similar standards as the RMSEA.

Finally, we investigated measurement invariance across age and sex groups by applying a multigroup analysis with theta parameterization. We divided participants into age groups of comparable sizes (≤39, 40–59, ≥59). For the analysis of invariance, we applied the χ^2^-test, the CFI, and the RMSEA. We treated a deviation of more than .01 in CFI and .015 in RMSEA between models as a sign that a measure is not invariant across the groups [50, 51]. Since the factor analysis method we employed treats our items as ordinal, we tested the following levels of invariance: configural (or pattern invariance, i.e., the number of factors and pattern of loadings is equal for both groups), metric (or weak; i.e., additionally the same magnitude of factor loadings across groups), threshold invariance (additionally the thresholds in the assumed latent response distribution are set to be equal across groups), and finally scalar invariance (or strong; additionally, the item intercepts are equated across groups). Finally, we used ω as a measure of factor score reliability [52].

## Results

### Item characteristics

Table 2 shows descriptive statistics such as means, standard deviations, along with values for skewness, and kurtosis for all CATS items, and its three scales. Furthermore, corrected item-total correlations are reported. As indicated by item difficulty indices and skewness values, the items of the *secure-scale* were evaluated in a positive manner (*P* = .39 to *P* = .69), whereas most participants disagreed with the statements proposed in the *avoidant-fearful-scale* (*P* = .25 to *P* = .53) and the *preoccupied-merger-scale* (*P* = .20 to *P* = .47). Internal consistency coefficients indicated good reliability for all three scales (ω_secure_ = .81; ω_avoidant-fearful_ = .75; ω_preoccupied-merger_ = .86). The final scale is illustrated in Table 4.

**Table 4:** Items of the final scale

**Secure**	
2. My counselor is sensitive to my needs.	
5. My counselor is dependable.	
14. When I show my feelings, my counselor responds in a helpful way.	
29. My counselor is a comforting presence to me when I am upset.	
**Avoidant fearful**	
3. I think my counselor disapproves of me.	
12. I don’t like to share my feelings with my counselor.	
15. I feel humiliated in my counseling sessions.	
18. Sometimes I’m afraid that if I don’t please my counselor, s/he will reject me	
**Preoccupied**	
4. I yearn to be “at one” with my counselor.	
7. I wish my counselor could be with me on a daily basis.	
13. I’d like to know more about my counselor as a person.	
25. I wish I could do something for my counselor too.	
Example of excluded items	
22. I wish there was a way to spend more time with my counselor.	
16. I think about calling my counselor at home.	
26. My counselor helps me to look closely at the frightening or troubling things that have happened to me.	

Item nr. as in the original CATS (Mallickrodt et al., 1995)

### Factor structure

In the initial PA, the empirical eigenvalues for the first three components were larger than the 95% confidence interval of the randomly generated ones (Table 5). Hence, it was confirmed that a three component should be extracted from the original matrix. We reported the factor loadings of the subsequent EFA in Table 2. Upon examination, the three-factor structure became evident.

**Table 5:** Eigenvalues from parallel analysis

	Empirical	Random
1	9.960	1.736
2	4.609	1.636
3	1.940	1.564
4	1.285	1.507
5	1.216	1.456

*PA* Parallel analysis; resulted in three factors

In Sample 1, the original model proposed by Mallinckrodt and colleagues [5] had moderate to unacceptable fit (see Table 6), which led us to seek for a better fitting model. To this end, we shortened the model based on item characteristics, as previously explained in the section of statical analyses. Items with low loadings (i.e., 7, 14, 17, 21, 24, 26, 31, 32, 36) and cross-loadings higher than .25 (i.e., 8,12) were excluded. In addition, items with item total correlation lower than .50 were discarded too (i.e., 7, 14, 22, 23, 26, 31, 32, 36) [42]. Finally, we used *stuart* on the remaining items. Among the possible 47,250 combinations, the algorithm selected the most suitable as indicated in Table 2. The aforementioned configuration was tested in Sample 2, showing excellent fit (Table 6). Correlations between the latent variables were small to moderate: *r*_*secure, avoidant-fearful*_ = −.363, *p* = .002, *r*_*secure, preoccupied-merger*_ = .232, *p* = .002, *r*_*avoidant-fearful, preoccupied-merger*_ = .164, *p* = .043.

**Table 6:** Confirmatory factor analysis results of the CATS (Samples 1 and 2)

Sample	Model	χ^2^(*df*)	*p*	CFI	TLI	RMSEA	SRMR
1	Original model	1821.859 (591)	<.001	.892	.885	.077	.101
1	Stuart / EFA	118.288 (51)	<.001	.974	.966	.061	.061
2	CFA	105.12 (51)	<.001	.955	.941	.060	.049

χ^2^ is Yuan-Bentler-scaled; *CMIN/DF* Minimum discrepancy divided by degrees of freedom; *CFI* Comparative fit index, *TLI* Tucker Lewis index, *RMSEA* Root mean square error of approximation, *SRMR* Standardized root mean square residua

#### Measurement invariance

To ensure the comparability of test results across different demographic groups, we computed measurement invariance across sex and age groups in the confirmatory sample (Study 2). As reported in Table 7, there is evidence for metric, threshold, and scalar factorial invariance across sexes. For age groups, we could also confirm metric and scalar invariance, but with regard to item response thresholds deviated slightly in terms of Δχ^2^ and Δ*CFI*, indicating non-equivalence for at least some of the thresholds. In contrast, a very low ΔRMSEA indicated invariance. By releasing equality constraints for the thresholds of Items 15 (“I think my counselor disapproves of me.”) and 18 (“I don’t like to share my feelings with my counselor.”), partial threshold invariance was attained.

**Table 7:** Fit indices for the analysis of measurement invariance - Sample 2

Model	χ^2^	*df*	Δ χ^2^	Δ*df*	Δ *p*	CFI	ΔCFI	RMSEA	ΔRMSEA
**Gender**									
Configural invariance	171.683	102				.974		.067	
Loading invariance	195.790	111	24.107	3	<.001	.968	.006	.070	.003
Threshold invariance	227.103	145	31.313	34	.600	.969	.001	.061	.009
Intercept invariance	222.572	154	4.531	9	.873	.974	.005	.054	.007
**Age groups** (≤39, 40–59, ≥59)									
Configural invariance	250.897	153				.967		.079	
Loading invariance	287.178	171	36.281	18	.006	.962	.005	.082	.003
Threshold invariance	386.929	231	99.751	60	.001	.949	.013	.082	.000
Partial threshold invariance ^a^	351.020	221	35.909	10	<.001	.957	.008	.076	.006
Intercept invariance	373.635	235	22.615	14	.067	.954	.003	.076	.000

*CFI* Comparative fit index, *RMSEA* Root mean square error of approximation

^a^ = The thresholds of Item 15 (“I think my counselor disapproves of me.”) and 18 (“I don’t like to share my feelings with my counselor.”) was freed to vary between groups

#### Validity

Convergent and discriminant validity were investigated using the HAQ, the BFKE, and the SCL-90. Zero-order correlations are reported in Table 8. To compare the predictive validity of the short scales to that of the original scales, we tested the correlations towards relevant constructs for significant differences. For the *secure-scales*, differences in *r* were never larger than .04, *z*s ≤ .53, *p*s ≥ .298. The avoidant-fearful-subscales differed with regard to their associations with two subscales: Patient-HAQ “relationship-satisfaction” (Δ*r* = .10, *z* = 1.752, *p* = .040) and BFCE “Readiness for Self-Disclosure” (Δ*r* = .12, *z* = 1.672, *p* = .047). These differences in *r* correspond to a *d* of .20 and .24, respectively, which are commonly interpreted as small effects. There were again no significant differences between correlations for the *preoccupied-merger* scales, Δ*r* ≤ .08, *z*s ≤ 1.08, *p*s ≥ .14. It should also be mentioned that the *secure*- and the *avoidant-fearful*-scale are correlated at *r =* −.56, compared to *r =* −.71 for the original CATS; Δ*r* = .15, *z* = 3.37, *p* < .001, *d* = .30.

**Table 8:** Correlations between the CATS and related psychological constructs (Sample 1)

	Secure^L^	Secure ^S^	Avoidant-fearful^L^	Avoidant-fearful ^S^	Preoccupied-merger^L^	Preoccupied-merger ^S^
Secure^L^	1					
Secure ^S^	.94*	1				
Avoidant-fearful^L^	−.71*	−.71*	1			
Avoidant-fearful ^S^	−.55*	−.56*	.87*	1		
Preoccupied-merger^L^	−.05*	−.02	.24*	.29*	1	
Preoccupied-merger ^S^	−.03	.00	.17*	.23*	.89*	1
HAQ-P RS	.74*	.75*	−.59*	−.49*	−.07	−.06
HAQ-P OS	.48*	.49*	−.41*	−.32*	−.12*	−.12*
HAQ-T RS	.31*	.31*	−.20*	−.15*	−.02	−.08
HAQ-T OS	.25*	.25*	−.21*	−.17*	−.11	−.12*
BFCE-FoR	−.24*	−.26*	.43*	.44*	.35*	.27*
BFCE-RfSD	.28*	.30*	−.42*	−.30*	−.06	−.03
BFCE-CNfC	−.11	−.11	.23*	.27*	.41*	.37*
SCL-GSI AN	−.09	−.12*	.23*	.24*	.25*	.19*
SCL-PST AN	−.06	−.10	.22*	.22*	.20*	.15*
SCL-GSI EN	−.28*	−.29*	.44*	.42*	.29*	.24*
SCL-PST EN	−.24*	−.25*	.40*	.36*	.28*	.24*

* = significant at an α-level of .05; ^L^ = scales according to Mallinckrodt et al. (1995); ^S^ = subscales according to the short model; HAQ RS = Relationship satisfaction; HAQ OS = outcome satisfaction; BFCE FoR = Fear of Rejection; BFCE RfSD = Readiness for Self-Disclosure; BFCE CNfC = Conscious Need for Care; SCL GSI = Global Severity Index; SCL PST = Positive Symptom Total

## Discussion

The CATS is widely used in psychotherapeutic research. However, the psychometric structure of the German adaption of the CATS has not yet been investigated, especially a confirmatory factor analysis is needed. This is of special importance, since patients were recently identified as “pseudosecure” attached patients due to a high intercorrelation between the clients’ secure attachment to the therapist (CATS-secure) with the clients’ avoidant attachment [27]. Therefore, the factorial structure was reevaluated in a large German-speaking sample and an item selection implemented in order to reduce this overlap of constructs.

Based on two large clinical samples, the present study demonstrated a poor fit for the CATS model proposed by Mallinckrodt and colleagues [5]. These results are consistent with and explicable by the high intercorrelation between the clients’ secure attachment to the therapist (CATS-*Secure*) with the clients’ avoidant attachment (CATS-*Avoidant-Fearful*; *r* = −.69, *p* < .001). In order to improve the model, items were eliminated based on relevant characteristics (item-total correlation, normal distribution of item scores, factor loadings). The resulting version of 12 items with 4/4/4 items per subscale showed a very good fit for this shortened model (see Table 6.). In addition, items that were eliminated also fit well the characteristic of the sample, being that our participants were inpatients. This implies that such setting represents a different type of answers and might reflect the attachment to the therapist in an inpatient environment, that might be experienced in a different way than outpatients. For example, item 16.: “I think about calling my counselor at home.” and item 22.: “I wish there was a way to spend more time with my counselor.” were excluded (Table 4). Since inpatients are constantly surrounded by the staff, either by nurses, doctors, social workers, coaches, or other room-patients the proximity seeking to the therapist is different than patients in an out-patient environment. Thus, the patient might not feel the same type of separation anxiety as an outpatient, who does not have this network of support in a daily basis.

Overall, evidence for invariance across sex is strong but for age groups it is weaker and should be reassessed in additional studies, es especially because we released equality constraints for the thresholds of two Items 15 (“I think my counselor disapproves of me.”) and 18 (“I don’t like to share my feelings with my counselor.”). Based on our data, we supposed that older people (≥59) compared to younger adults (≤39) would have different attitudes toward their counselor. From the perspective of developmental and personality psychology, we assumed that older adults would have stricter judgement toward their therapist give that they tend score higher on neuroticism [53] compared to younger people.

This economic version also showed a good and improved reliability, compared to the original model proposed by Mallinckrodt and colleagues [5] with reliability values of Alpha = .81/64/.63. Based on this empirical approach, a questionnaire was gained with a good model fit. In addition, an economic version was generated, which is well applicable in the clinical settings with patients with psychological symptoms. This model was also invariant for sex and age groups.

The present study showed very little disparity between long version and short version of the CATS. In addition, the convergent validity for the new short version is given as well. This could be shown based on the convergent validity to the Helping Alliance Questionnaire (HAQ) as well as another questionnaire measuring the expected attachment to the therapist (BFCE). Specifically, clients who scored low on the insecure BFCE-scales (*Fear of rejection, Conscious need of care*), high on the secure BFCE-scales *Readiness of self-disclosure* scored also high on the *CATS Secure* subscale. As in the present data, these patients reported positive working alliances and tend to perceived their therapists as emotionally responsive, accepting, and promoting a “secure base” for exploration (e.g., “I feel that somehow things will work out OK for me when I am with my counselor”) [1, 54, 55]. They show a greater self-disclosure [56] and high in-session exploration [15]. It has been shown that this type of behavior strengthens the working alliance [27, 57, 58]. A strong working alliance is related to improvement in symptoms, global functioning, and interpersonal problems [59]. In this context it is worth mentioning that validity depends on making strong assurances of anonymity, as originally stated by Mallinckrodt and colleagues (1995). The authors mentioned that their participants were assured that their therapist or counselors would never have access to their assessment of the counseling relationship. Such information is crucial for the patients in order to feel comfortable and truly share their internal state with their therapist without hesitation. Otherwise, patients could question the anonymity of their data and be reluctant, which may compromise the validity of the scale.

Clients who scored high on the insecure BFCE- scales, low on the secure BFCE- scales, *Readiness of self-disclosure* also scored high on the CATS *Preoccupied-Merger* subscale and desired more frequent and intensely personal contact and long to be “at one” with the therapist. Clients with these characteristics tend to be highly willing to depend on the therapist, fear rejection, abandonment, and wish to be their therapist’s “favorite” client [5, 60]. The correlations of the CATS *Preoccupied-Merger* subscale with working alliance suggest a poor working alliance characterized by a bond without agreement about the therapy’s goals, as previously observed [15, 61]. According to [62] this type of behavior is related to an insecure attachment, which reflects in behavioral hyperactivation (e.g., a heightened need for proximity and desire to blur professional boundaries: “I think about calling my counselor at home”). Especially adults with a background of dysfunctional interactions with family members, memories of emotionally unavailable parents tend to develop this type of attachment [63]. Clients who scored high on the insecure BFCE- scales, low on the secure BFCE- scales *Readiness of self-disclosure* also scored high on the CATS *Avoidant-Fearful* subscale. These patients distrusted their therapists, showed fear of rejection and were reluctant with self-disclosure and reported the poorest working alliances, as previously evidenced [27, 64, 65]. It has been explained that avoidant patients typically decline requests to express their emotions and feel discomfort when getting closer to the therapist [60, 61]. In addition, the high correlations between the psychological symptom questionnaire (SCL-90R) and the *avoidant-fearful* as well as *preoccupied-merger* scale of the CATS showed that greater attachment insecurity is associated with greater psychological symptoms, which is consistent with a large body of literature [12, 66, 67].

Even though this study is based on relatively large samples of patients, there are several limitations to be considered. For example, there are differences between the original sample [5] (sample a) and our sample (sample b), that could explain why the factor analyses differed: (1) Sample b), were all inpatients, while the participants in sample a were all outpatients. (2) Sample a) had one therapist and this person was the exclusive focus of ratings; Sample a) worked with many individuals in the inpatient setting (3) Sample a) completed ratings early in the first 5–6 sessions treatment, whereas the participants in our study completed the CATS near termination after about 12 sessions and more than 60 days of continuous inpatient treatment.

Finally, the original CATS is based on an English-speaking population, while our sample is based on German-speaking participants, which may culturally differ from one another. Furthermore, we were not able to collect detailed sociodemographic variables other than age and sex in the second study (Sample 2). However, this matter did not affect any of the statistical analyses. Moreover, no interaction between therapist adult attachment and patient attachment to therapist could be investigated since the adult attachment style of the therapist was not assessed. The importance of such interactions was highlighted by Petrowski and colleagues [24], who found that therapist’s insecure adult attachment was associated with more insecure client attachment to therapist. Moreover, patient attachment to therapist was assessed only by a self-report in the present study. The effect of patient attachment to therapist should be investigated by different methods. In future studies, the patients’ representation of the therapists’ using the Patient-Therapist Adult Attachment Inventory (PT AAI) by Diamond and colleagues [68] might be promising. The PT-AAI [68, 69], is a semi-structured interview developed as an adaptation of the AAI aimed at classifying the mental state concerning patients’ attachment to their therapists, and vice versa.

In general, the short version of the German-version of the CATS showed very good fit. Importantly, we included items that have moderate difficulty in order to maximize sensitivity across the broadest range of the trait. However, it is possible that extreme values cannot be capture with this short version.

For future research, the short version of the CATS has to be implemented in a large clinical sample in order to replicate the psychometric properties and the factorial structure of the CFA. Also, there are still numerous unanswered questions such as, for example, how counter-complementary attachment behavior can and should be used in therapeutic settings [70]. For clinical practice, it would be of interest whether pre-treatment and possible earned security status of the patients would further influence the therapeutic process. In addition, the therapy drop-out rate as well as disorder specific effects needs to be examined more closely in reference to the clients’ attachment to the therapists. Further research on the interaction processes between the attachment representations of the patients and the therapists assessed by using the same measures should be carried out with respect to the therapeutic outcome.

## Conclusions

The present study investigated a German adaptation of the original CATS and proposed a shortened version of with improved validity. The CATS-S will be helpful in properly identifying types of client attachment, and we recommend it for medical and psychological research.

## Data Availability

The dataset used and analyzed during the current study is available from the corresponding author on reasonable request.

## Abbreviations

CATS: Client Attachment to Therapist Scale
SCL: Symptom Check List
BFCE: Bielefeld Client Expectations Questionnaire
HAQ: Helping Alliance Questionnaire
AAI: Adult Attachment Inventory
CMIN/DF: Minimum discrepancy divided by degrees of freedom
CFI: Comparative Fit Index
TLI: Tucker Lewis Index
RMSEA: Root Mean Square Error of Approximation
SRMR: Standardized Root Mean Square Residual
BIC: Bayesian Information Criterion
GH: Gamma hat
